# Impaired intrinsic functional connectivity between the thalamus and visual cortex in migraine without aura

**DOI:** 10.1186/s10194-019-1065-1

**Published:** 2019-12-19

**Authors:** Heng-Le Wei, Xin Zhou, Yu-Chen Chen, Yu-Sheng Yu, Xi Guo, Gang-Ping Zhou, Qing-Qing Zhou, Li-Jie Qu, Xindao Yin, Junrong Li, Hong Zhang

**Affiliations:** 1 Department of Radiology, The Affiliated Jiangning Hospital of Nanjing Medical University, No.169, Hushan Road, Nanjing, Jiangsu Province, 211100 China; 2 Department of Neurology, The Affiliated Jiangning Hospital of Nanjing Medical University, No.169, Hushan Road, Nanjing, Jiangsu Province, 211100 China; 3 Department of Radiology, Nanjing First Hospital, Nanjing Medical University, Nanjing, Jiangsu Province, 210006 China

**Keywords:** Migraine, Thalamus, Visual network, Functional magnetic resonance imaging

## Abstract

**Background:**

Resting-state functional magnetic resonance imaging (fMRI) has confirmed disrupted visual network connectivity in migraine without aura (MwoA). The thalamus plays a pivotal role in a number of pain conditions, including migraine. However, the significance of altered thalamo-visual functional connectivity (FC) in migraine remains unknown. The goal of this study was to explore thalamo-visual FC integrity in patients with MwoA and investigate its clinical significance.

**Methods:**

Resting-state fMRI data were acquired from 33 patients with MwoA and 22 well-matched healthy controls. After identifying the visual network by independent component analysis, we compared neural activation in the visual network and thalamo-visual FC and assessed whether these changes were linked to clinical characteristics. We used voxel-based morphometry to determine whether functional differences were dependent on structural differences.

**Results:**

The visual network exhibited significant differences in regions (bilateral cunei, right lingual gyrus and left calcarine sulcus) by inter-group comparison. The patients with MwoA showed significantly increased FC between the left thalami and bilateral cunei and between the right thalamus and the contralateral calcarine sulcus and right cuneus. Furthermore, the neural activation of the left calcarine sulcus was positively correlated with visual analogue scale scores (*r* = 0.319, *p* = 0.043), and enhanced FC between the left thalamus and right cuneus in migraine patients was negatively correlated with Generalized Anxiety Disorder scores (*r* = − 0.617, *p* = 0.005).

**Conclusion:**

Our data suggest that migraine distress is exacerbated by aberrant feedback projections to the visual network, playing a crucial role in migraine physiological mechanisms. The current study provides further insights into the complex scenario of migraine mechanisms.

## Introduction

Migraine is typically characterized as a throbbing, unilateral pain and is usually accompanied by nausea, vomiting, and exaggerated sensitivities to normally well-tolerated light, noise and smell [1]. Migraine patients often suffer from anxiety, depression, sleep disturbances, and other comorbid conditions, significantly increasing the social burden and decreasing the quality of daily life [2]. Approximately one-third of migraineurs are preceded by visual, auditory, or somatosensory symptoms, termed the aura. The most common type of aura is characterized by visual discomfort which is associated with a reversible, transient and depolarized or hyperpolarized cortical event, termed cortical spreading depression (CSD). In addition, approximately 90% of migraineurs report symptoms of light hypersensitivity during a migraine attack, and about 45% report these symptoms in the interictal period [3, 4]. Therefore, investigating the mechanisms of the visual network in migraine may have significant implications for our understanding of the pathophysiology of migraine and its prognosis.

In recent decades, the pathophysiological mechanisms of visual pathway alterations have been extensively explored in neural disorders using different experimental approaches. Resting-state functional magnetic resonance imaging (fMRI) has proven to be a noninvasive and useful technique to explore the underlying pathogenesis of migraine-induced neural dysfunction, and spontaneous blood oxygenation level-dependent (BOLD) responses have been used to probe the structural and functional abnormalities likely to contribute to migraine [5, 6]. However, functional imaging studies have not yet reached a consensus on the relevant changes in brain activation of the visual cortex. In terms of migraineurs with aura, some studies have demonstrated stronger activation in many brain regions associated with sensory-discriminative regulation, cognitive processing and pain modulation [7, 8]. On the other hand, some studies about migraineurs without aura have shown conflicting abnormalities compared with healthy controls (HC) [9, 10]. With the independent component method, migraine was linked to disrupted resting-state functional connectivity (FC) in multiple intrinsic neural networks including the visual network [11]. Although migraine has been characterized as a neurodevelopmental disorder of brain dysfunction, the pathophysiological mechanisms between aberrant activation of the visual network and emotional symptoms is still unclear.

Furthermore, previous functional imaging studies have proven that the thalamus, a key component of the trigemino-thalamo-cortical pathway, is regarded as a relay region for transmitting information out to the cerebral cortex and receiving feedback information from the cerebral areas [12, 13]. CSD is a potential neurophysiological phenomenon underlying the migraine and resulting in cortical network depolarization [4]. CSD-associated nociceptive information is transmitted through the trigeminovascular system to the brainstem and subsequently to thalamic and cortical areas to produce the sensation of pain [14]. In addition, some studies have demonstrated abnormal thalamic FC with the visual network or visual-related cortex in migraineurs compared with HC [15, 16]. With the diffusion tensor imaging technique, the study showed significantly higher fractional anisotropy and lower mean diffusivity in the bilateral thalami in migraine patients without aura [17]. These neurophysiological studies have already demonstrated that thalamic anomalies produce an alteration in the cortical processing of sensory information, including visual information. Notably, migraine and migraine-induced symptoms have a strong association with anxiety and depression [2]. Additional network circuits have been implicated in migraine pathophysiology and other types of negative emotional and affective processes, including the thalamo-cortical system, based on evidence for altered microstructures and functional connectivities [18]. Nevertheless, few of the currently available studies have specifically revealed abnormal thalamic FC with the visual network and correlations with neuropsychiatric symptoms. Therefore, probing the mechanisms of abnormal FC of the thalamus with the visual network and neuropsychiatric symptoms in MwoA may have significant implications for our understanding of migraine pathophysiology and its prognosis.

## Materials and methods

### Subjects

Thirty-three consecutive, right-handed episodic migraine patients were prospectively recruited from our hospital. The inclusion criteria were in accordance with the International Classification of Headache Disorders, Third Edition (ICHD-3. Code 1.1) [19]. Twenty-two age- and sex-matched, right-handed subjects with no family history of migraine, were recruited from hospital staff members and their relatives. The exclusion criteria were the following: chronic systemic diseases, illness affecting central nervous system function, substance abuse, or contraindications to MRI. All patients were in the interictal state, the time from the end of the last attack being at least 72 h, while an interval of at least 72 h from the next attack, ascertained by a telephonic interview. Written informed consent was obtained from all participants according to the approval of the ethics committee of our university.

### Assessments and neuropsychological tests

Demographic data included age, sex and the following clinical characteristics obtained from migraineurs without aura: disease duration, mean pain intensity of migraine attacks, attacks frequency, duration of attacks and related psychological tests. The mean pain intensity of migraine attacks was measured using a visual analogic scale (VAS). Headache impact was obtained using the Headache Impact Test-6 (HIT-6). Moreover, measures of anxiety and depression were obtained using the Generalized Anxiety Disorder-7 (GAD-7) and the Patient Health Questionnaire-9 (PHQ-9), respectively.

### MRI scanning parameters

MRI data were acquired using a 3.0 T MRI scanner (Ingenia, Philips Medical Systems, Netherlands) with an 8-channel receiver array head coil. The participants were instructed to lie quietly with their eyes closed and think of nothing, but to remain awake. To improve image quality, earplugs and foam pads were used to attenuate scanner noise and minimize head movements. Structural images were acquired with a three-dimensional turbo fast echo T1WI sequence with high resolution as follows: repetition time (TR) = 8.1 ms; echo time (TE) = 3.7 ms; slices = 170; thickness = 1 mm; gap = 0 mm; flip angle = 8°; matrix = 256 × 256; and field of view (FOV) = 256 mm × 256 mm. The structural sequence took 5 min and 29 s. Functional images were acquired axially using a gradient echo-planar imaging sequence as follows: TR = 2000 ms; TE = 30 ms; slices = 36; thickness = 4 mm; gap = 0 mm; FOV = 240 mm × 240 mm; matrix = 64 × 64; and flip angle = 90°. The fMRI sequence took 8 min and 8 s.

### Data preprocessing

Standard image data preprocessing, statistical analysis and visualization were performed by a toolkit from Data Processing Assistant for Resting-State fMRI (DPARSF; http://www.restfmri.net). The first 10 points of all subjects were discarded to avoid unstable magnetization. Afterwards, the remaining images were processed by the following steps: slice-timing adjustment, realignment, spatial normalization into Montreal Neurological Institute (MNI) (resampling voxel size = 3 mm × 3 mm × 3 mm), smoothing with a 4-mm Gaussian kernel, detrending and filtering (0.01–0.08 Hz). The participants who had head motion less than 2.0 mm displacement or a 2.0° rotation in any direction were included. To control for non-neural noise in the time series, parameters for head motion, white matter (WM) signal, and cerebrospinal fluid (CSF) signal were included as covariates in the linear regression.

Structural data were preprocessed using the Statistical Parametric Mapping software (SPM8, http://www.fil.ion.ucl.ac.uk/spm) and a voxel-based morphometric (VBM) analysis. Images were first segmented into gray matter (GM), WM and CSF partitions. Total brain parenchyma volume was computed as the sum of GM and WM volumes. Subsequently, the GM partitions were utilized to create a template using the diffeomorphic anatomical registration through exponentiated lie algebra (DARTEL) algorithm. The warped GM images were then modulated and resliced within the template. Finally, a Gaussian kernel with a full-width at half-maximum (FWHM) of 8 mm was used to smooth all the GM images. With respect to the region of interest (ROI) volume, the thalamus was defined using WFU PickAtlas software (http://www.ansir.wfubmc.edu). Obvious structural damage was not observed based on the conventional MRI series.

### Independent component analysis (ICA) and ROI-wise FC analysis

Imaging data after preprocessing were analysed by Group ICA of fMRI Toolbox (GIFT, http://mialab.mrn.org/software/gift/). The number of independent components was determined using the fastICA and the self-organizing group ICA algorithms. The GICA back reconstruction step was used to separate single-subject components from the set of aggregate components calculated by the previous step. The visual network was selected from these components as the “best-fit” network component, previously described in an earlier fMRI study [20]. To estimate intra-group spatial consistency, we performed one-sample *t*-tests (*p* < 0.05, family-wise error (FWE) corrected) on the visual network spatial maps. To estimate inter-group differences in the visual network, we used two-sample *t*-tests (*p* < 0.001, uncorrected) within the mask created by the one-sample *t*-test, and the covariates (age, sex and GM volume) were removed to control their effects. The surviving clusters were reported and extracted as ROIs. Finally, individual ICA Z-scores were extracted from the visual clusters identified in the above analyses and used for linear correlation analyses with clinical parameters and neuropsychological test results.

The thalamus ROI was generated by WFU_PickAtlas software. Then, Pearson’s correlation coefficients were used to calculate ROI-wise FC matrices between the mean time series of the thalamus and that of each ROI extracted from the visual network above. Finally, Fisher’s transform was conducted to normalize the correlation coefficients. For between-group comparisons, the statistical inference was performed at *p* < 0.001, uncorrected. Age, sex, whole-brain GM volume and ipsilateral thalamus volume were included as nuisance covariates.

### Statistical analysis and correlation analysis

Differences in demographic data were analysed using a between-group *t*-test for means and a *Chi-square* test for proportions; *p* < 0.05 was significant. To examine the relationships between signal changes in neural activation and clinical indicators of MwoA, the mean Z-values of surviving regions identified by ICA and FC analysis were extracted for each patient. Partial correlations were implemented for calculating the correlation between the mean Z-values and clinical indicators after adjusting for age, sex, GM volume and ipsilateral thalamic parenchyma volume using SPSS 24.0 (version 24.0; SPSS, Chicago, IL, USA), and the *p* value of less than 0.05 was considered statistically significant.

## Results

### Demographic data and structural MRI findings

Table 1 summarizes the demographic and neurophysiological characteristics of the participants who were included in this study. The patients with MwoA and HC did not show significant differences in terms of age or sex. Moreover, the volumes of both groups did not reveal any significant differences, neither at a statistical threshold corrected for multiple comparisons (FDR corrected, *q* < 0.05) nor at an uncorrected threshold (*p* < 0.001; cluster size > 100) (Table 2).

**Table 1:** Characteristics of patients with MwoA and healthy contrasts

	Patients with MwoA (*n* = 33)	Healthy controls (*n* = 22)	*p* value
Age (year) ^a^	36.06 ± 10.58	32.86 ± 7.20	0.188
Gender (male/female)	5/28	5/17	0.498
Duration of disease (year) ^b^	8 (2, 13)	/	/
Frequency (times/month) ^b^	3 (3, 4)	/	/
Duration of attacks (hour) ^b^	12 (8, 15)	/	/
HIT-6 score ^a^	58.94 ± 9.28	/	/
VAS score ^a^	6.52 ± 1.35	/	/
GAD-7 score ^b^	4 (2.5, 9)	/	/
PHQ-9 score ^b^	5 (3, 9.5)	/	/

^a^Data are presented as mean ± SD. ^b^Data are reported as medians and interquartile ranges (25th–75th percentiles). HIT-6: Headache Impact Test-6; VAS: visual analogue scale; GAD-7: Generalized Anxiety Disorder-7; PHQ-9: Patient Health Questionnaire-9; MwoA: migraine without aura

**Table 2:** Comparisons of volumes between patients with MwoA and healthy controls

Brain region (cm^3^)	Patients with MwoA (n = 33)	Healthy controls (n = 22)	*p* value
Left thalamus	5.98 ± 0.40	6.05 ± 0.53	0.575
Right thalamus	5.93 ± 0.39	5.97 ± 0.52	0.753
Bilateral thalami	11.91 ± 0.80	12.02 ± 1.05	0.660
Gray matter	626.34 ± 48.66	647.57 ± 62.10	0.162
White matter	480.74 ± 45.89	479.03 ± 50.59	0.897
Cerebrospinal Fluid	213.22 ± 19.79	210.54 ± 17.23	0.607
Brain parenchyma	1107.08 ± 87.73	1126.60 ± 104.06	0.456

Data are presented as mean ± SD. MwoA: migraine without aura

### ICA results

**Fig. 1:**
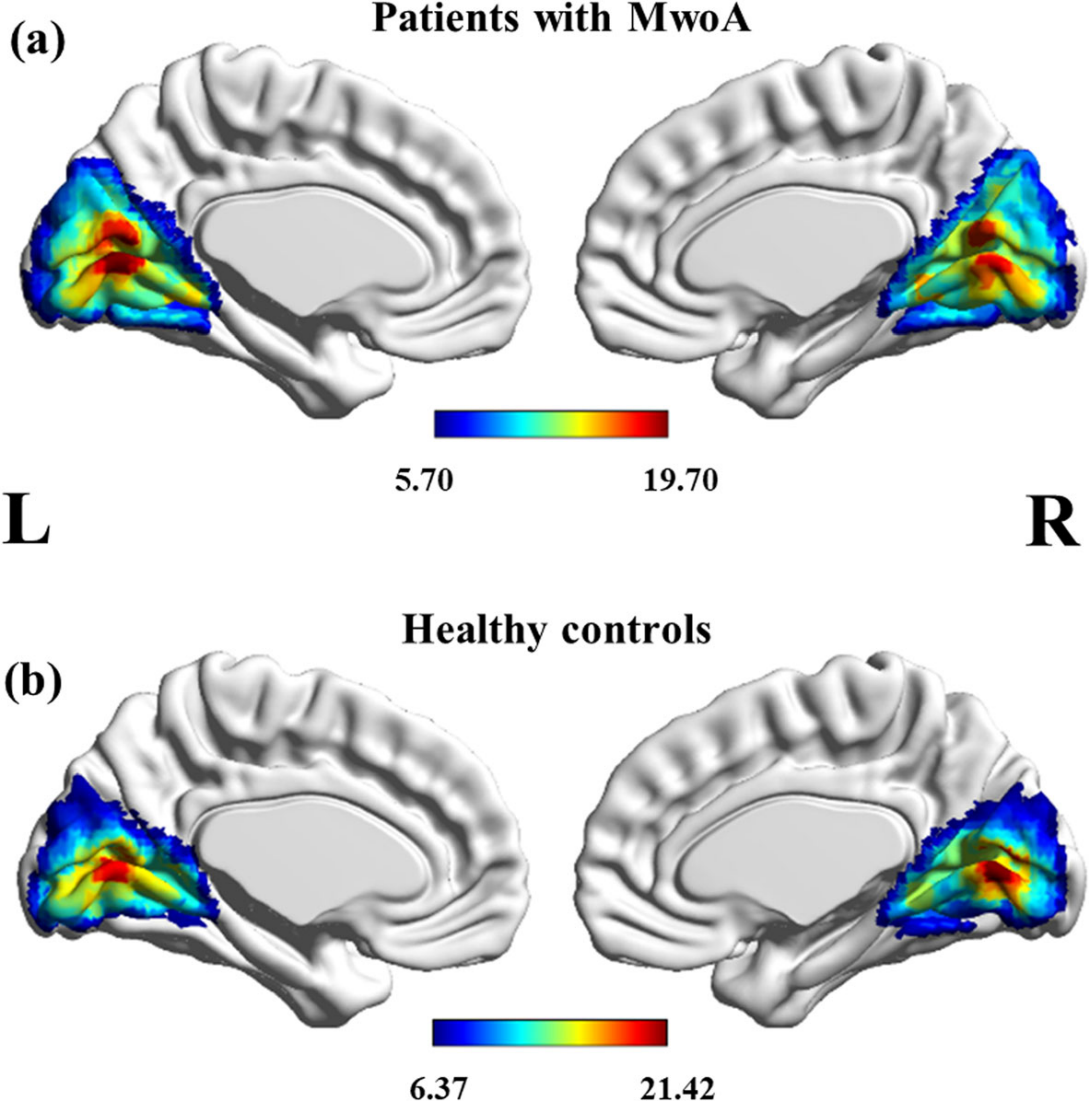
Group-level visual network in patients with MwoA (**A**) and healthy controls (**B**). Statistical maps were overlaid on the inflated 3D brain surface from the ‘Colin 27’ atlas. Significant thresholds were corrected using cluster-level family-wise error (FWE) correction and set at *p* < 0.05, cluster size > 30. MwoA: migraine without aura

**Fig. 2:**
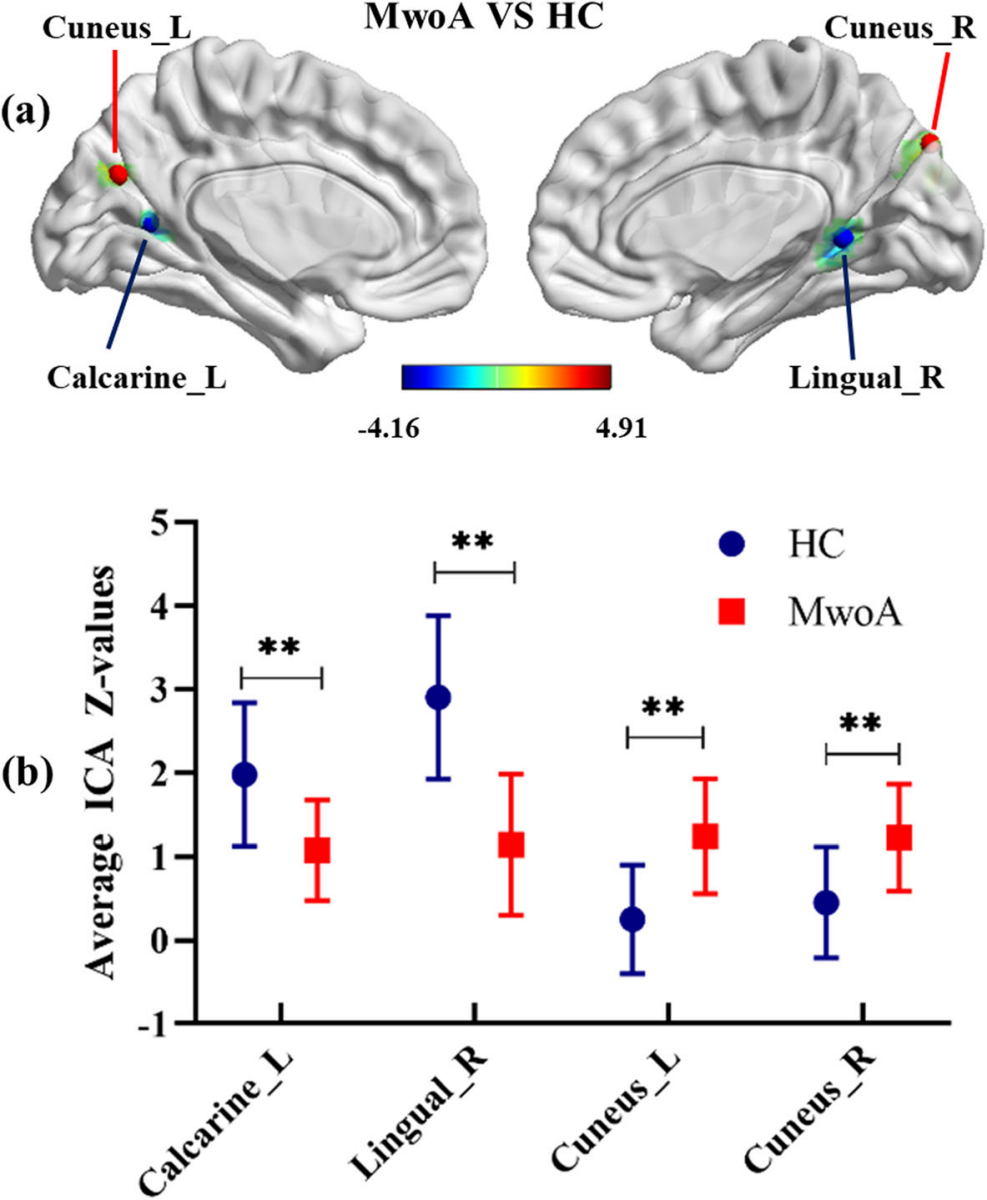
T-maps of statistically significant differences within the visual network between patients with MwoA and HC (*p* < 0.001, uncorrected) (**A**). Comparison of average ICA Z-values in surviving visual areas between patients with MwoA and HC (**B**). ICA: independent component analysis; MwoA: migraine without aura; HC: healthy controls; L: left; R: right; **p < 0.001

As illustrated in Fig. 1, a resting-state visual network, encompassing certain occipital and temporal cortices, corresponded to the previous description in both migraine patients and HCs. The resting-state visual network exhibited statistically significant regional differences between the two groups (*p* < 0.001, uncorrected). The bilateral cunei had an increased component activity in patients with MwoA compared with HC. However, the right lingual gyrus and left calcarine cortex demonstrated the opposite neural activity in MwoA relative to controls (Fig. 2; Table 3). Specifically, the two-sample *t*-tests revealed significant differences in the ICA Z-values of the significant brain regions in Fig. 2 (*p* < 0.001).

**Table 3:** Significant differences in ICA-Z value within visual areas between two groups

Brain region	Peak MNI coordinates (x, y, z)			Voxels size	Peak intensity
Cuneus_L	-6	− 72	27	17	4.3309
Cuneus_R	15	−84	39	16	3.9559
Calcarine_L	−24	−60	9	18	−4.1568
Lingual_R	15	−51	3	16	−3.9999

The threshold was set at *p* < 0.001 (uncorrected). *ICA* independent component analysis; *MNI* Montreal Neurological Institute; *L* left; *R* right

### ROI-wise FC analysis and correlation analysis results

**Fig. 3:**
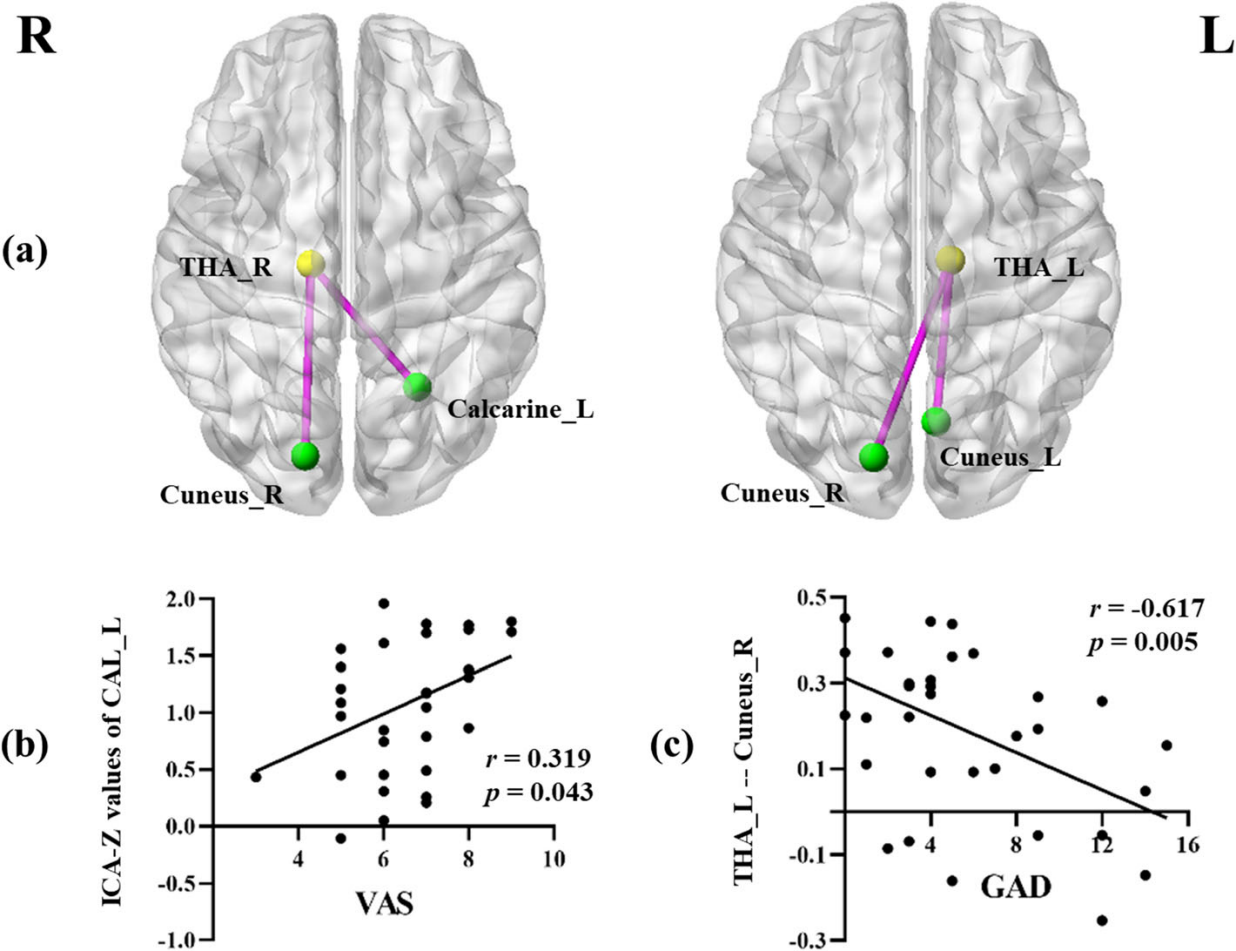
Aberrant ROI-wise functional connectivity between the bilateral thalami and visual network in patients with MwoA compared with HC (**A**). The threshold was set at *p* < 0.001, uncorrected. The significant positively correlation between the ICA-Z values of the left calcarine sulcus and VAS scores (**B**). The significant negatively correlation between functional connectivity of the left thalamus to the right cuneus and GAD scores (**C**). THA: thalamus; CAL: calcarine; ICA: independent component analysis; VAS: visual analogic scale; GAD: generalized anxiety disorder; L: left; R: right

Compared with HC, patients with MwoA showed significantly increased connectivity between the left thalamus and bilateral cunei. Moreover, the right thalamus also showed a stronger connectivity with the contralateral calcarine cortex and right cuneus (Fig. 3). The threshold was set at *p* < 0.001, uncorrected (cluster size > 10).

Furthermore, in patients with MwoA, the decreased ICA values of the left calcarine sulcus were positively correlated with VAS scores (*r* = 0.319, *p* = 0.043). For the left thalamus, the enhanced FC to the right cuneus demonstrated a negative correlation with GAD scores (*r* = − 0.617, *p* = 0.005) (Fig. 3). None of the other aberrant FC values were correlated with GAD or PHQ scores.

## Discussion

In the current study, we compared GM, WM and brain parenchyma volumes but did not detect any differences, which was consistent with our previous study [21]. However, previous studies have reported disrupted volume measures in migraine patients in several brain networks, including the visual network [22, 23]. A longitudinal analysis [23] demonstrated that GM volume changes in migraine are dynamic and remodelled over time according to patients’ clinical features, and that many regions may be involved in the regulation of the progression of migraine characteristics. Since we did not detect any significant differences in GM volume between migraine patients and HC, the most likely explanation for this might be the absence of classification of clinical features in migraine patients, such as pain severity, disease duration and attacks frequency. On the other hand, an alternative possibility is that analytical techniques were not sensitive enough to detect regional differences in GM volume or intensity. Our findings suggested that abnormal neural activity and functional networks may exist prior to major structural alterations in patients with MwoA.

Consistent with previous network analyses, this study showed that several brain areas exhibited significantly abnormal ICA values, including the cuneus, lingual gyrus and calcarine sulcus. These areas are the major regions of the visual network identified in previous resting-state positron emission tomography (PET) [24] and fMRI [25] studies. ICA values decreased in the right lingual gyrus as well as in the left calcarine sulcus, which are mainly involved in processing and modulation of pain [26, 27]. A resting-state fMRI study [28] showed that similarly decreased regional homogeneity of the lingual gyrus in MwoA with long-term and short-term disease duration, compared with HC, and showed that a long history of MwoA might contribute to accumulating brain dysfunction due to repetitive attacks. Furthermore, PET research [29] has illustrated hypermetabolism in the right lingual gyrus, which is involved in visual-related perceptual abnormalities (e.g., photophobia, afterimages and visual snow) and non-visual symptoms (e.g., tinnitus). However, our findings did not detect any correlation between abnormal neural activation or volume of the lingual gyrus and scores from the neuropsychological tests. From the above findings, we speculated that resting-state functional abnormalities involving the lingual gyrus play a crucial role in migraine physiological mechanisms, but share certain modulation processes in different migraine subtypes and comorbidities.

In addition, decreased ICA values of the left calcarine sulcus were positively correlated with VAS scores in patients with MwoA. The calcarine sulcus, located on the medial surface of the occipital lobe, is the main node within the visual network. Moreover, the calcarine sulcus is responsible for multisensory processing in affective, sensory and cognitive aspects of pain [3, 30]. Furthermore, abnormal FC of the calcarine has been shown to be involved in many independent networks, corresponding to the perspective that the experience of pain is complex and involves multidimensional processing [31]. Thus, the lower activity of the left calcarine sulcus in MwoA patients could be related to the lower tolerance threshold to the normally noxious or non-noxious sensory stimuli. Besides, a longitudinal investigation [23] showed a significant morphological correlation between migraine progression and visual areas, especially in the calcarine cortex and cuneus. Our findings may indirectly explain the regulation of the calcarine sulcus in headache severity in another aspect. We speculated that the decreased ICA values of the left calcarine sulcus in patients with MwoA could be related to functional impairments in pain compensatory mechanisms and aggravate the visual burden in the long-term pain response.

The cuneus is within the extrastriate cortex and is involved in visual selective attention by relaying top-down information from the attention network to visual areas [32]. One PET study [33] showed that in patients with migraine, activation of the primary visual cortex was induced by the same luminous stimulation, during the spontaneous headache and after pain relief. Although the activation of the cuneus was captured in both conditions, no cortical activation was found in the attack-free period as expected by the similar luminous stimulation, which is contrary to our results. However, another PET study [24] did not induce significant activation in controls, but there was greater activation within the visual cortex in interictal migraineurs, with constant uniform luminance stimulus. Clinically, it has been hypothesized that migraineurs with visual discomfort have a lower sensitivity threshold to visual stimulation visually and a decreased pre-activation level of the sensory cortex compared with healthy subjects. The presentation of our results, in terms of the cuneus when there is increased activation in the interictal period, may account for the disrupted neural habituation. Therefore, the higher activation of the bilateral cunei may be involved in a compensatory role in the deficit of habituation and relieving the headache.

However, regional brain dysfunctions alone are not sufficient to explain the pathological mechanisms of migraine. Recent resting-state fMRI studies have provided evidence that dysfunctional connectivity within pain pathways and other sensory pathways led to the development of migraine during or between the attacks [25, 34]. The perception of nociceptive signals is mediated by the thalamus and the thalamic neurons project signals to somatosensory and visual cortices depending on the trigeminal pathway [35]. Most importantly, we provided direct evidence that spontaneous BOLD fluctuations in FC between the left thalamus and the right cuneus were negatively correlated with anxiety in the resting state. Our results are similar to recent resting-state fMRI findings in which the bilateral thalami participate in the regulation of the visual pathway [36]. In the current study, heightened intrinsic connectivity within the visual network in migraineurs may thereby set the stage for abnormally intensified responses to sensory information, such as pain-related signals. Increased connectivity of the thalamus to the visual cortex could provide a neuroanatomical framework for understanding why visual stimuli that are well-tolerated in controls can elicit an unpleasant experience in migraineurs and evoke escape responses. One possible explanation was that the pathogenesis of migraine seems to be driven by complex dysfunction of thalamic FC and temporal activation of neuronal networks. If the feedback system that inhibits visual perception is dysfunctional, the aberrant visual signals will be passed on to the cortex causing the conscious perception of nociception. Taken together, these studies suggest that the thalamus might regulate the balance facilitation and inhibition within dysfunctional pain control centers and plays a crucial role in modifying the top-down activity of pain control processing in migraine.

From the aforementioned discussions, the thalamus is a core structure in transmitting sensory input to the cortex, including painful and other negative signals. Our data indicate the involvement of thalamo-visual connectivity in the modulation of pain severity. This is in line with the effective preventative medications in the thalamus, such as beta-blockers and gabapentin [37, 38]. Additionally, migraine is more than just headache, and we have to take into account that migraineurs are suffering from accompanying symptoms to various sensory stimuli, even those that are well-tolerated by healthy controls. Our findings further suggest that neuronal modulation of the thalamo-visual pathway is responsible for emotional processing, such as anxiety, and decreases the adaptation of the thalamic cortex to negative signals. However, a recent study highlighted the regulatory mechanisms of brainstem circuits, rather than the thalamus as a core source of sensory adaptation [39].

The present study has several limitations. First, we did not remove the global signal in order to avoid spurious negative correlations. The negative correlations among brain regions have been associated with global signal removal, and this removal may affect correlation analyses between the thalamus and the visual cortex. Second, we must admit that no significant results persisted after the use of FDR correction, probably due in part to the relatively strict calculation. A more stringent threshold and *Bonferroni* correction will be considered in future studies. Nonetheless, our research is still meaningful for providing some insight in this field. Third, the difference in the timing of the data collection is another limitation in this study. Migraineurs can be studied during or between attacks, and migraineurs in different phases and subtypes have been induced different neural functional results. This limitation should be taken into account when interpreting the resting-state study, and this study might reflect only the neuropathological mechanisms of a particular subtype of migraine to some degree. Fourth, FC method could potentially help improve our understanding of underlying migraine mechanisms, but so far it has been suspected to be not reproducible and no reproducible neuroimaging biomarkers of migraine have been identified [40]. Finally, in addition to aberrant FC patterns, more studies are required to explore the possibility of differences in structural connectivity within the visual network in patients with MwoA, which can be measured by diffusion tensor imaging.

## Conclusion

Despite these limitations, our current study identified associations of abnormal thalamic FC patterns to the visual network with anxiety in migraineurs without aura. In addition, disrupted neural activation of visual areas was detected in patients with MwoA in the resting state. These findings mainly explicate the possible role of the potential neural interactions within the thalamo-visual pathway, which may lead to a better understanding of the pathophysiology of MwoA.

## Data Availability

Clinical, neuroimaging and statistical data will be available upon request from any qualified investigator.

## Abbreviations

BOLD: Blood oxygenation level dependent
FC: Functional connectivity
fMRI: Functional magnetic resonance imaging
GAD: Generalized anxiety disorder
HC: Healthy controls
HIT: Headache impact test
ICA: Independent component analysis
MwoA: Migraine without aura
PHQ: Patient health questionnaire
VAS: Visual analogic scale
